# Diet in Disease

**Published:** 1893-08-19

**Authors:** Ernest Hart

**Affiliations:** Bachelier-ès-Sciences-es-Lettres (rèstreint), formerly Student of the Faculty of Medicine of Paris, and of the London School of Medicine for Women


### DIET IN DISEASE.

By Mrs. Ernest Hart, Bachelier-ès-Sciences-es-Lettres (rèstreint), formerly Student of the Faculty of Medicine of Paris, and of the London School of Medicine for Women.

### XXVIII.—CHRONIC BRIGHT'S DISEASE.

If my readers will turn to the chapter in which the structure of the kidney was described, it will be found that that organ was represented as performing in the body the part of a scavenger and a filter. It will be remembered that the arterial blood is brought by the short arteries of the kidney straight from the main current into a close tangle of loops of blood vessels called a glomerulus. This glomerulus is pushed into the blind and expanded head of a long uriniferous tubule. The cells lining this bag-like end of the tubule are small and flat, and they act merely as a filter. The blood, carried with considerable force straight from the renal artery, is brought to a sudden condition of stasis in the loops of the glomerulus, and it is easily understood how, under these circumstances, the blood parts with a good deal of its fluid. What, however, passes from the blood is not its nutritive constituent parts, but its excess of water and the excretory products with which it is laden. These products are particularly urea and urates, which are, as has been already fully explained, the final products of the digestion of albuminous foods.

Now, if instead of the blood parting only with its, urea and water in the kidney, it allowed the albumen, which forms its most important nutritive constituent, to pass across the filter into the uriniferous tubules, and, if at the same time the excretory products of urea and urates were retained in the blood, an abnormal highly prejudicial to health condition would be present, for there would be an extravagant waste of albumen, out of which the blood reconstructs the tissues and fluids of the body, and the blood instead of being cleansed in the kidney would remain charged with the poisonous products of combustion. This is what occurs in chronic Bright's disease. The kidney is damaged; it fails to separate the urea from the blood, and it allows the albumen to escape into the tubes. The consequences are an increasing weakness and frequently emaciation, an enfeeblement of the mental powers owing to the presence of effete products in the blood, a thickening of the arteries due to the irritative action of these products on the walls of the arteries, a consequent embarrassment of the action of the heart, venous congestion, and the escape of the fluid of the blood into the tissues, with the consequent symptoms of dropsy.

There are obviously, therefore, three important considerations to be thought of in treating chronic Bright's disease, namely, how to prevent the waste of albumen, how to make good this waste, and how to prevent poisoning by the retention of urea and effete products in the blood. Before, however, considering treatment it would be as well to inquire into the

**Causes of Bright's Disease.**—These are acute fevers, cold, alcohol, and excessive eating of meat.

Acute albuminuria is an almost constant symptom in scarlet fever, and frequently of the other eruptive fevers. In these cases recovery from the albuminuria is with appropriate treatment the rule, but sometimes the attacks leave behind a certain enfeeblement of the action of the kidney, which may finally result in the establishment of chronic Bright's disease. The progress of the disease may be so slow and insidious that it is not noticed till certain unexplained symptoms lead to the examination of the urine, when albumen is found to be present. Cold is a frequent cause of Bright's disease in those cabmen, whose work requires them to be exposed to all weathers. The abuse of alcohol also frequently results in chronic albuminuria. The presence of albumen in the blood seems to exercise a peculiarly irritating influence on the tissues of the kidney. To excessive consumption of meat, and the consequent labour thrown upon the kidneys, some of the cases of Bright's disease, from which the well-to-do and well fed suffer, are attributed. Worry and anxiety are also, there is little doubt, frequently the cause of chronic albuminuria. It may be objected to the assertion regarding meat eating as a cause that a great variety of experiments have demonstrated that temporary albuminuria cannot be produced experimentally by an excessive albuminous diet. This may be the case, and yet a continuous use of an extravagant albuminous diet, combined at the same time with an abuse of alcohol, may and does throw on the kidney so great a work of elimination, that sub-acute congestion and consequent albuminaria may be the result. Certain it is that in meat eating countries Bright's disease is much more prevalent than in those where the staple articles of diet are rice and fish.

**Treatment by Diet.**—Two objects must be aimed at: to diminish the loss of albumen, and the production of effete excretory products. It has been erroneously thought that because albumen is lost by the kidney, that there must be an excess of albumen in the diet to compensate for this loss; but it is forgotten that while the albumen is lost by the blood the urea is retained, and that as in the digestion of albumen, urea is the final product, an excess of albumen in the food, will, when the kidneys do not excrete all the urea, lead to a dangerous accumulation of poisonous products in the blood, with probably uræmic poisoning, coma, and death as the result. The indications therefore are to give such nutritious food as will maintain the strength without increasing nitrogenous refuse in the blood. It has been found that the best food in these cases of Bright's disease is milk, and if it can be well borne the happiest results have been obtained from an exclusive milk diet. It is quite remarkable how beneficial the result often is when a rigid milk diet has been maintained. The amount of urine has increased, the albumen excreted has diminished, the amount of urea has been augmented, the dropsy has disappeared, and the improvement in strength and general well-being has been most marked. The milk should be taken as fresh as possible, and in small quantities at short intervals, namely, about six ounces every hour during the day, one glass on getting up and another on going to bed. In all about from three to four quarts a day should be taken. If the milk is disliked and produces a disagreeable taste in the mouth, it can be mixed with a little lime water or aërated water, or a small amount of alkaline water can be taken after the glass of milk. If the milk treatment is tolerated by the patient, it should be continued either till there is a complete disappearance of albumen from the urine, or till the amount of it is very much reduced. This period varies in different cases, but usually after six to eight weeks of milk diet there is such a marked improvement in the condition of the patient, and such a striking amelioration of symptoms, that he may be allowed to gradually return to a mixed diet. If there should afterwards be a return of the dropsy and albuminuria the milk diet should be again enforced.

